# Supplementary material for: Polyketide synthase gene clusters in the ericoid mycorrhizal fungus Oidiodendron maius: genome-wide prediction, expression profiling, and functional characterization of knockout mutants
Source: Front Plant Sci. 2026 Sep 16;17:1924430. doi: 10.3389/fpls.2026.1924430 (PMC13623903; doi:10.3389/fpls.2026.1924430)
Supplement: Supplementary file 1 [file SupplementaryFile1.zip › Supplementary Materials/Supplementary Folder 1.zip!/Oidma1_AssemblyScaffolds_Repeatmasked/knownclusterblast/cluster57/jgi.p_Oidma1_107059_mibig_hits.html]

| MIBiG Protein | Description | MIBiG Cluster | MiBiG Product | % ID | % Coverage | BLAST Score | E-value |
| --- | --- | --- | --- | --- | --- | --- | --- |
| ABW71833.1 | putative\_aldehyde\_dehydrogease | BGC0000303 | Anthramycin\_biosynthetic\_gene\_cluster | 33 | 96.3782696177 | 228 | 1.6e-59 |
| ABB90277.1 | aldehyde\_dehydrogenase | BGC0001057 | Zearalenone\_biosynthetic\_gene\_cluster | 31 | 100.60362173 | 212 | 9.3e-55 |
| CAQ52616.1 | benzaldehyde\_dehydrogenase | BGC0001066 | Kendomycin\_biosynthetic\_gene\_cluster | 34 | 90.7444668008 | 206 | 8.7e-53 |
| AAY93391.1 | phenylacetaldehyde\_dehydrogenase\_PeaE | BGC0000413 | Pyoverdine\_biosynthetic\_gene\_cluster | 31 | 96.9818913481 | 199 | 8.2e-51 |
| BAP16694.1 | aldehyde\_dehydrogenase | BGC0000376 | JBIR-34,\_JBIR-35\_biosynthetic\_gene\_cluster | 30 | 92.7565392354 | 190 | 6.5e-48 |
| AFT91402.1 | hypothetical\_protein | BGC0000338\_c2 | Echinocandin\_B\_biosynthetic\_gene\_cluster | 28 | 93.5613682093 | 186 | 5.5e-47 |
| ABK32267.1 | AmbN | BGC0000014 | Ambruticin\_biosynthetic\_gene\_cluster | 28 | 99.7987927565 | 183 | 4.6e-46 |
| AEF33093.1 | putative\_aldehyde\_dehydrogenase | BGC0001039 | Pyridomycin\_biosynthetic\_gene\_cluster | 28 | 93.7625754527 | 175 | 1.6e-43 |
| XP\_011392748.1 | hypothetical\_protein | BGC0001281 | Ustilagic\_acid\_biosynthetic\_gene\_cluster | 25 | 89.939637827 | 119 | 8.2e-27 |
